# Metabolic Syndrome and Abnormal Peri-Organ or Intra-Organ Fat (APIFat) Deposition in Chronic Obstructive Pulmonary Disease: An Overview

**DOI:** 10.3390/metabo10110465

**Published:** 2020-11-15

**Authors:** Niki Katsiki, Anca Pantea Stoian, Paschalis Steiropoulos, Nikolaos Papanas, Andra-Iulia Suceveanu, Dimitri P. Mikhailidis

**Affiliations:** 1 First Department of Internal Medicine, Diabetes Center, Division of Endocrinology and Metabolism, AHEPA University Hospital, 1st Stilponos Kyriakidi, 54621 Thessaloniki, Greece; nikikatsiki@hotmail.com; 2 Diabetes, Nutrition and Metabolic Diseases Department, “Carol Davila” University of Medicine, 8 Eroii Sanitari, 050474 Bucharest, Romania; 3 Department of Pneumonology, Democritus University of Thrace, 68100 Alexandroupolis, Greece; pstirop@med.duth.gr; 4 Diabetes Centre, Second Department of Internal Medicine, Medical School, Democritus University of Thrace, 68100 Alexandroupolis, Greece; 5 Faculty of Medicine, Ovidius University, 900470 Constanta, Romania; andrasuceveanu@yahoo.com; 6 Department of Clinical Biochemistry, Royal Free Hospital campus, University College London Medical School, University College London (UCL), London NW3 2QG, UK; mikhailidis@aol.com

**Keywords:** chronic obstructive pulmonary disease, metabolic syndrome, metabolic markers, non-alcoholic fatty liver disease, epicardial fat, non-alcoholic fatty pancreas disease, intramuscular fat, abnormal peri-organ fat, abnormal intra-organ fat

## Abstract

Chronic obstructive pulmonary disease (COPD) is a common disorder with an increasing prevalence, characterised by persistent respiratory symptoms and airflow limitation. Systemic inflammation is involved in the pathogenesis of COPD and can also predispose to metabolic disorders (e.g., metabolic syndrome (MetS) and non-alcoholic fatty liver disease (NAFLD)). Such comorbidities can negatively affect COPD outcomes, cardiovascular risk, and quality of life. Apart from NAFLD, abnormal peri-organ or intra-organ fat (APIFat) could be considered as markers for cardiometabolic diseases and even for COPD. The present narrative review considers the associations of COPD with MetS, NAFLD, and other APIFat, including epicardial, perirenal, peripancreatic, and intramuscular adipose tissue. Further research is needed to define these relationships and identify any potential clinical implications.

## 1. Introduction

Chronic obstructive pulmonary disease (COPD), mainly caused by tobacco smoking, is characterised by persistent respiratory symptoms and airflow limitation [1]. However, increasing evidence indicates that COPD has important extra-pulmonary manifestations, resulting in significant systemic abnormalities [2,3]. Whether these abnormalities represent direct consequences of the pulmonary disorder, or whether COPD is, in fact, a systemic disease, is still a matter of debate [4].

A central feature in the pathogenesis of COPD includes an impaired inflammatory response of the lungs to noxious particles or gases, caused by an imbalance between oxidant and antioxidant factors; this results in a local increase in oxidative stress and inflammation [5]. The hypothesis that systemic inflammation originates by “spill over” from the pulmonary compartment to the systemic circulation is still to be proven [6]. Genetic and constitutional factors may also predispose individuals with COPD towards the development of systemic and pulmonary inflammation [7]. Systemic inflammation is present in patients with stable COPD, as demonstrated by the increase in serum levels of acute phase proteins, i.e., C-reactive protein (CRP), fibrinogen, interleukins (IL-6 and IL-8), and tumour necrosis factor α (TNF-α), as well as the increased numbers of circulating leucocytes [8]. Additionally, inflammatory biomarkers correlate with an increased risk of COPD exacerbations [9]. There is also increasing evidence supporting that oxidative stress is involved in the development and progression of COPD [10].

Systemic inflammation plays a key role in the pathogenesis of COPD systemic effects, including weight loss, skeletal muscle dysfunction, and cardiovascular (CV) complications [2]. On the other hand, inflammation and oxidative stress have also been implicated in the development of metabolic disorders such as the metabolic syndrome (MetS) and non-alcoholic fatty liver disease (NAFLD) [11,12,13,14]. Both diseases are associated with an increased CV risk [15]. Apart from NAFLD, research has been also extended to the links between other abnormal peri-organ or intra-organ fat (APIFat) deposits and cardiometabolic disorders [16,17].

The aim of the present narrative review was to discuss the associations of COPD with MetS, NAFLD, and APIFat at different locations (namely epicardial, perirenal, peripancreatic, and intramuscular adipose tissue).

## 2. MetS and COPD

An association between MetS and COPD has been reported [18,19]. Briefly, MetS was present in 62% of 76 COPD patients, whereas COPD was diagnosed in 22% of 59 MetS patients [18]. In another study [19], MetS prevalence was 37.8% among 98 COPD patients. Increased rates and duration of acute COPD exacerbations have been related to the presence of MetS [20]. In addition to increasing the levels of inflammatory markers, MetS has been associated with limited physical activity in patients with COPD, independently of their lung dysfunction [21]. Furthermore, the circulating levels of leptin, endothelin, CRP, and IL-6 were significantly higher, whereas levels of adiponectin were significantly lower, in patients with both COPD and MetS compared with COPD patients without MetS [22,23]. Higher serum leptin levels were associated with greater systemic and airway inflammation in patients with stable COPD [24]. Of note, increased circulating leptin levels have also been observed in obese patients [25] and patients with type 2 diabetes mellitus (T2DM) [26,27]. Furthermore, adiponectin concentrations have been positively related with COPD severity and progression [28] and increased respiratory mortality in COPD patients [29]. Leptin and adiponectin levels may vary according to COPD phenotypes (e.g., in relation to emphysema development and progression) [30] and their circulating levels may be affected by statins and antidiabetic drugs (metformin, pioglitazone, empagliflozin and liraglutide) [31,32,33]. Overall, inflammation, adipokine dysregulation, smoking and physical inactivity may contribute to the link between MetS and COPD [34]. Smoking was also shown to increase the expression of pro-inflammatory mediators by perivascular fat tissue [35].

Among the MetS diagnostic components, dyslipidaemia (i.e., low high-density lipoprotein cholesterol and high triglyceride levels), fasting hyperglycaemia, hypertension, and abdominal obesity (expressed as increased waist circumference) were inversely related with lung function; waist circumference was the strongest predictor of lung dysfunction [36]. Abdominal obesity, hyperglycaemia, and hypertension may represent the most prevalent MetS components found in COPD patients [37,38].

The presence of MetS increases the risk of CV morbidity and mortality in several conditions, including COPD [39,40,41,42]. Therefore, it has been suggested that COPD patients should be screened for MetS [43,44] not only due to the possible co-existence of these disorders, but also because MetS treatment may improve COPD prognosis [45]. In this context, the presence of MetS was associated with increased dyspnoea and co-morbidities (such as DM, heart failure, coronary artery disease, and osteoporosis) in COPD patients hospitalised for an exacerbation [46].

In the context of MetS and COPD, there is a need to consider the “obesity paradox” (i.e., evidence that obesity in patients with COPD is associated with a reduced all-cause mortality) [47]. This pattern is also seen in some other diseases [47]. Several factors are probably involved in mediating this unexpected relationship. These have been reviewed elsewhere [47] and are beyond the scope of the present review. Furthermore, we need to consider the role of “reverse causality”. In other words, that pathological weight loss rather than weight gain contributes to the “obesity paradox” [47].

Overall, it is still not clear whether MetS could be a direct consequence of the COPD-related progressive lung dysfunction, in the absence of cigarette smoking or exposure to air pollution [48]. However, chronic systemic inflammation, adipose tissue dysfunction, oxidative stress, inhaled and oral glucocorticoids therapy, physical inactivity, hyperglycaemia, and aging could be involved in the development of MetS and other metabolic disorders in COPD patients [48].

**Figure 1:**
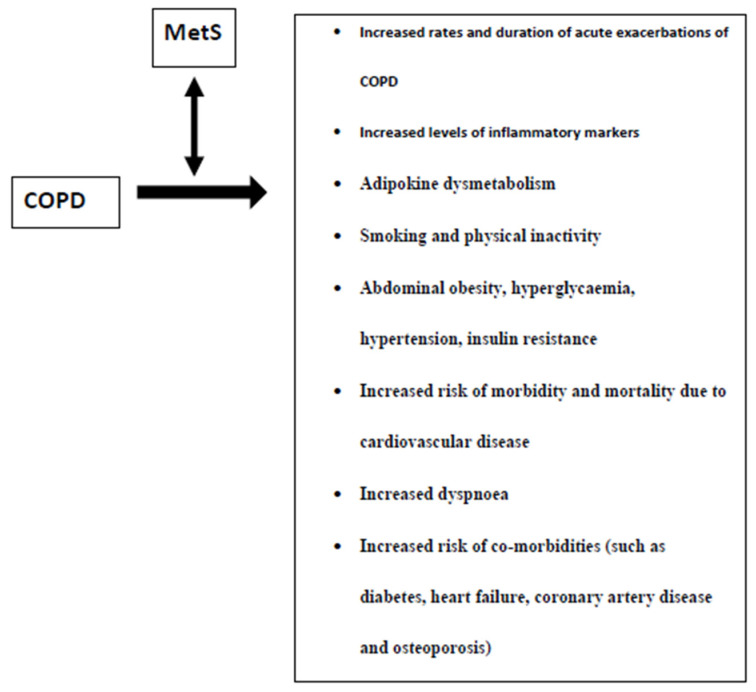
Characteristics of the presence of the metabolic syndrome (MetS) in patients with chronic obstructive pulmonary disease (COPD).

Figure 1 summarizes the characteristics of MetS presence in COPD patients. Since the presence of MetS has been linked to COPD severity as well as increased CV risk in COPD patients, further research is needed in this field to elucidate the importance of preventing/treating MetS in improving the prognosis of COPD patients.

## 3. NAFLD and COPD

NAFLD, a hepatic manifestation of MetS, is characterised by fat accumulation in the liver in the absence of alcohol abuse or other causes of chronic liver disease [49]. NAFLD can progress from benign steatosis to steatohepatitis, cirrhosis, and, potentially to hepatocellular carcinoma [50]. Inflammation, oxidative stress, and insulin resistance play a key role in NAFLD pathophysiology [51,52]. NAFLD is associated with increased liver and CV morbidity and mortality [40,53,54,55]. Furthermore, NAFLD patients have a 2-fold increased risk of developing T2DM [56,57]. The prevalence of NAFLD is very high in T2DM patients reaching almost 60% as reported in recent meta- analyses [58,59].

NAFLD was also highly prevalent in COPD patients [60]. Briefly, among 111 COPD patients, the prevalence of liver steatosis, non-alcoholic steatohepatitis and liver fibrosis was 41.4, 36.9, and 61.3%, respectively [60]. Furthermore, NAFLD presence and severity were associated with pulmonary dysfunction [61,62,63]. Vice versa, reduced lung function [defined by forced expiratory volume in 1 s (FEV_1_) and forced vital capacity (FVC)] was a risk factor for NAFLD incidence in a large (*n* = 96,104) middle-aged (mean age 35.7 years) healthy Korean cohort [64]. This bilateral relationship between NAFLD and pulmonary function has also been confirmed in a recent meta-analysis [65].

Statins and antidiabetic drugs may beneficially affect NAFLD [66,67,68,69,70,71]. An expert panel considered the use of pioglitazone and statins for NAFLD treatment [72]. Statins can also improve lung function, mortality, and exacerbation rates in COPD patients possibly due to their anti- inflammatory properties [73,74]. In this context, a previous meta-analysis found that statins significantly decreased all-cause mortality (HR: 0.65, 95% confidence intervals (CI), 0.57–0.74, *p* < 0.001) and COPD exacerbation rates with or without hospitalisation (HR: 0.58, 95% CI, 0.48–0.72, *p* < 0.001) [75]. In other meta-analyses, statins improved pulmonary function and exercise tolerance in COPD patients [76] and decreased COPD mortality [77]. Statins may also protect against pulmonary arterial hypertension (PAH) secondary to lung diseases [78,79]. A recent meta-analysis found that statins significantly reduced all-cause and coronary heart disease mortality, acute exacerbation of COPD, CRP, and PAH in COPD patients [80].

It has been suggested that statin-induced benefits in COPD patients could be more pronounced in those patients with cardiometabolic co-morbidities compared with those without a CV indication for statin use [81]. However, data from randomised controlled studies are missing [82,83]. Further research is needed to elucidate the role of statins in the treatment of COPD.

We also need to consider the role of subcutaneous adipose tissue (SAT) and abdominal visceral adipose tissue (VAT), as well as liver fat. In this context, a study found that, COPD patients (*n* = 1,267) with prior myocardial infarction (MI) had a significantly greater VAT area than those with no history of MI [84]. All fat deposits were evaluated using computed tomography. However, there was no such relationship between a history of MI and SAT or liver attenuation [84]. Furthermore, after adjusting for several factors (including obesity and diabetes), COPD patients in the upper tertile VAT area had an increased odds ratio (1.86; 95% CI 1.02–3.41) of MI compared with patients in the lower tertiles [84].

Overall, the role of NAFLD on COPD development and progression should be further evaluated in order to improve patients’ prognosis.

## 4. Epicardial Fat and COPD

Epicardial fat thickness (EFT) has been associated with NAFLD, MetS, and CV disease [85,86,87]. In this context, a recent meta-analysis reported that EFT was significantly increased in NAFLD patients compared with controls (standardized mean difference 0.73, 95% CI 0.51–0.94; *p* < 0.001); elevated EFT was also related to NAFLD severity and CV disease in NAFLD patients [88]. Similarly, a previous meta-analysis found a significant correlation between EFT and MetS features (i.e., systolic blood pressure, waist circumference, triglycerides, high-density lipoprotein cholesterol, and fasting blood glucose); *p* < 0.0001 for all comparisons [89]. Increased EFT was also shown to predispose coronary heart disease (CHD) patients to sudden cardiac death [90]. Furthermore, a prospective population-based study (*n* = 4093; mean follow-up: 8 years) reported that increased EFT was associated with the incidence of both fatal and non-fatal coronary events [91]. Briefly, EFT doubling was related to a 1.5-fold risk of coronary events (HR 1.54, 95% CI 1.09–2.19; *p* < 0.001) [91]. From a pathophysiological point of view, EFT can exert both local and systemic harmful effects via the secretion of reactive oxygen species and inflammatory and proatherogenic cytokines [92]. Therefore, both inflammation and oxidative stress are involved in the increased CV risk linked to EFT [93,94].

In patients with CV disease (COPD patients were excluded), visceral obesity was associated with an adverse adipokine–cytokine profile [95]. In the same study, epicardial adipose tissue thickness was associated with perivascular adipose tissue [95].

The association between EFT and COPD has been investigated in a small number of studies. Sova et al. [96] found that COPD patients (*n* = 157) had a significantly higher EFT than controls (*n* = 45) (5.4 ± 1.6 vs. 4.1 ± 0.9 mm; *p* < 0.001); multivariate analysis showed that EFT was independently correlated with high-sensitivity CRP (β = 0.300; *p* < 0.001) and BODE index, an important prognostic predictor of COPD (β = 0.405; *p* < 0.001) [97]. The BODE index includes body mass index (B), degree of airflow obstruction (O), dyspnoea (D), and exercise capacity (E) [98]. Similarly, in an older study, EFT of COPD patients (*n* = 171) was significantly higher than controls (*n* = 70) (143.7 vs. 129.1 cm^3^; *p* = 0.02) and was correlated with CRP (*r* = 0.32; *p* < 0.001) and coronary calcium score (*r* = 0.38; *p* < 0.001) [99].

Likewise, COPD patients (*n* = 82) also had a significantly increased EFT than controls (*n* = 84) (6.1 ± 0.9 vs. 4.8 ± 1.1 mm; *p* < 0.001), as well as COPD patients with MetS compared with those without MetS (7.7 ± 1.8 vs. 6.1 ± 0.9; *p* < 0.001) [100]. In the same study, each 1 mm increase in EFT resulted in a 2-fold increase for MetS [100]. Furthermore, a positive relationship was found between EFT and the apnoea–hypopnea index (AHI) in 162 COPD patients (β = 0.10; *p* < 0.001); while continuous positive airway pressure (CPAP) therapy for 24 weeks significantly decreased EFT (from 7.29 ± 1.27 to 6.92 ± 1.26 mm; *p* = 0.005) [101]. Interestingly, EFT was also reported to be significantly associated with FEV_1_ (% predicted), FEV_1_/FVC and airway wall thickness [102].

On the other hand, there are studies with contradictory results. EFT was found to be decreased in COPD patients (*n* = 98) compared with healthy controls (*n* = 40) [103]. COPD patients with severe right ventricular systolic dysfunction (RVSD) also had a thinner EFT compared with those with mild RVSD [93]. Another study reported no differences in EFT between COPD patients (*n* = 81) and controls (*n* = 81) [104]. This diversity in findings may be explained, at least partly, by differences in patient characteristics, duration, and severity of COPD, as well as in the differences in the applied methodology of EFT measurements.

Currently, there is a knowledge gap in relation to the clinical implications of changes in EFT in COPD patients. This should be addressed in terms of identifying potential benefits for treating these patients.

### 4.1. Perirenal Fat and COPD

There is evidence of a link between perirenal adiposity and CV disease [105]. Associations between perirenal fat and other fat depots (e.g., NAFLD, EFT) have also been reported [106]. The presence of perirenal adipose tissue in COPD patients has not been investigated yet.

### 4.2. Peripancreatic Fat and COPD

Non-alcoholic fatty pancreas disease (NAFPD) has been related to CV disease [107]. Links between NAFPD and NAFLD, as well as EFT have also been reported [108,109,110]. It has been hypothesized that COPD may contribute to NAFPD development through systemic and local inflammation, excessive lipolysis and increased lipid synthesis, as well as intermittent hypoxia- induced pancreatic beta cell damage, thus representing potential mechanisms linking COPD with NAFPD [111]. Insulin resistance has also been related to COPD [112,113], as well as to NAFPD [114]. However, no data exist on peripancreatic adipose tissue in relation to COPD.

### 4.3. Intramuscular Fat and COPD

COPD is characterized by gradual skeletal muscle wasting, contributing to impaired exercise capacity, reduced health-related quality of life, and increased mortality [115].

The aetiology of muscle weakness/atrophy in patients with COPD is complex [116]. Cytokines, oxidative stress, hormones, nutrition, and obviously, decreased physical activity are likely to be involved [116]. This topic has been reviewed elsewhere [116].

Accumulation of adipocytes and intramyocellular lipid species in skeletal muscle can lead to loss of muscle strength and reduced insulin sensitivity [117]. The mechanisms involved in causing “myosteatosis” have not been well documented but are likely to involve aging, glucocorticoid treatment, estrogen deficiency, and disuse atrophy [117]. Inflammation and oxidative stress may also be involved in intramuscular fat accumulation [118,119].

Robles et al. reported significantly higher fat infiltration in calf and thin muscles in 10 COPD patients compared with controls, in addition to the reported muscle atrophy [120]. Of note, intramuscular fat infiltration was more profound than muscle atrophy in COPD patients [120]. Similarly, the percentage of intramuscular adipose tissue was higher in COPD patients (*n* = 101) than in healthy controls (*n* = 10) (6.7 ± 3.5 vs. 4.3 ± 1.2%; *p* = 0.03); this fat depot was associated with exercise capacity and physical activity level, thus potentially affecting COPD-related muscle dysfunction [121]. Furthermore, muscle fat infiltration was linked to oxidative stress and endothelial dysfunction in both obese and lean COPD patients (*n* = 78) [122]. In another study, intramuscular adipose tissue of knee extensors and flexors was 2-fold higher in COPD patients (*n* = 21) than healthy individuals (*n* = 21) [123]. It was recently reported that in patients with advanced emphysema, bronchoscopic lung volume reduction treatment was associated with increases in intramuscular fat [124]. The associations between intramuscular adiposity and COPD severity/prognosis should be further elucidated.

In the context of treatment, we should consider the potential adverse effects of statins on muscles [125,126,127] in COPD where there may already be impaired muscle function, as mentioned above [115,120].

## 5. Current Knowledge and Future Perspectives

**Figure 2:**
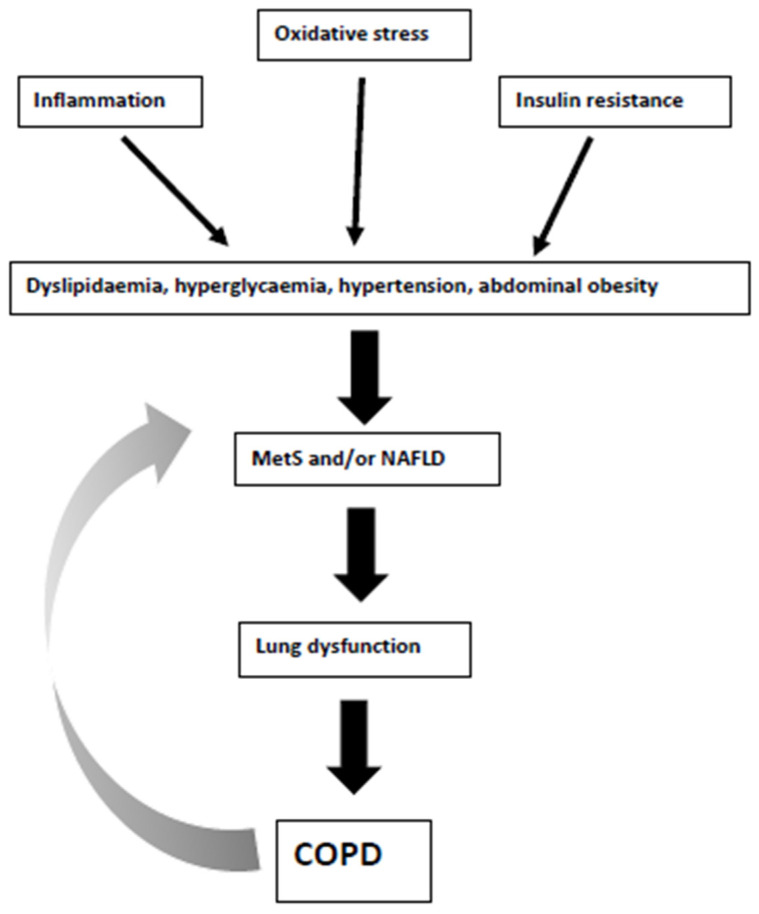
Pathophysiological links between metabolic syndrome (MetS), non-alcoholic fatty liver disease (NAFLD) and chronic obstructive pulmonary disease (COPD). The grey arrow shows the potential bilateral interactions between MetS/NAFLD and COPD.

COPD may represent a “systemic” disease with inflammation, oxidative stress and insulin resistance playing key roles as the underlying mechanisms linking COPD with cardiometabolic disorders. Among the latter, MetS and NAFLD have been related to the presence and severity of COPD, as well as with increased CV morbidity and mortality [18,20,40,54,60,63]. In this context, dyslipidaemia, hyperglycaemia, hypertension, and abdominal obesity (i.e., features of both MetS and NAFLD) are predictors of lung dysfunction [36]. In other words, the pathogenesis of COPD and MetS has been described as “intertwined” [48]. Figure 2 summarizes the pathophysiological links between MetS, NAFLD, and COPD. On the other hand, treatment of MetS and NAFLD may improve survival and COPD prognosis [45,72]. Therefore, screening COPD patients for MetS and NAFLD may result in early diagnosis and treatment, leading to better outcomes.

Apart from NAFLD, other APIFat deposits (i.e., epicardial, perirenal, peripancreatic, and intramuscular) have been related to cardiometabolic disorders [16,17,128]. Furthermore, we should consider that excessive “orthotopic” APIFat fat accumulation may be a “systemic” rather than a “local” phenomenon. Therefore, the reported associations of these fat depots with COPD may also be relevant. In this context, EFT has been found increased in COPD patients, being also linked to inflammation (i.e., CRP levels), COPD prognosis and severity (i.e., the BODE index, FEV_1_, FEV_1_/FVC, and airway wall thickness) [102]. However, as mentioned previously, there are also conflicting results [103,104]. Perirenal and NAFPD have been associated with CV risk, NAFLD, and EFT [106,108,109,110], but there are no data on their association with COPD, despite the common pathogenetic mechanisms (e.g., oxidative stress, inflammation, and insulin resistance). Future research on this field is certainly needed.

Finally, intramuscular fat infiltration may be more profound than muscle atrophy in COPD patients [120], thus potentially affecting their exercise capacity and physical activity level [121]. A link between intramuscular adiposity and lung function has also been reported in patients with advanced emphysema [124]. The associations of intramuscular fat with COPD should be further investigated.

Statins may improve both biochemical and histological features of NAFLD, as well as pulmonary function, exercise tolerance, (all-cause and CV) mortality, and exacerbation rates in COPD patients [72,73,75,80]. However, strong evidence from randomised controlled studies is lacking and further research should focus on elucidating the role of statins in COPD treatment.

The Overlap syndrome (OS) is characterised by the coexistence of COPD and obstructive sleep apnoea syndrome (OSAS) in the same patient [129]. Increasing evidence now acknowledges the frequent association between OSAS and MetS [130,131] as well as between OSAS and NAFLD [132,133]. Increased EFT has also been reported in OSAS patients compared with controls, even in the absence of obesity [134,135]. Interestingly, EFT has been linked to OSAS severity [136]. In this context, EFT was the strongest predictor of AHI among other parameters (i.e., age, gender, BMI, carotid intima-media thickness, and left ventricular mass index) in patients with both MetS and OSAS [137].

Overall, it has been suggested that OSAS may predispose to APIFat depositions via intermittent hypoxia, hormonal derangements, systemic and local inflammation, excessive lipolysis, and enhanced lipid synthesis [111]. OSAS severity has also been positively associated with adipose accumulation in skeletal muscles, a link that may be largely mediated by obesity [138].

Continuous positive airway pressure (CPAP) therapy was shown to reduce EFT [101]. No data exist on perirenal and peripancreatic fat deposition in OSAS patients.

Patients with OS exhibit worse parameters of nocturnal hypoxia, as well as frequent daytime hypoxaemia and hypercapnia, associated with pulmonary hypertension. In this context, hypoxia-inducible factor 1-alpha levels may be associated with tissue hypoxia in patients with COPD [139]. In turn, this factor may contribute to cardiac fibrosis and impaired cardiac function [139].

Furthermore, COPD and OSAS may share the same risk factors including smoking, obesity, inflammation (both systemic and local) as well as increased airway resistance [140]. Of note, OS patients may also have an increased CV morbidity compared with patients with OSAS alone [141,142,143]. These additive effects of concurrent COPD and OSAS point to an increased prevalence of comorbidities, particularly cardio-metabolic [144,145]. In this context, the presence of abnormal APIFat deposits in patients with OSAS and OS merits further research.

## 6. Conclusions

COPD patients may have an increased risk of developing MetS, NAFLD, increased EFT, and intramuscular fat. One should also consider that excessive “orthotopic” APIFat accumulation may be a “systemic” rather than a “local” phenomenon. These comorbidities may affect COPD progression as well as individual CV risk and also should be considered as a marker for developing cardiovascular disease and evolution for COPD. The role of peripancreatic and perirenal adipose tissue in COPD patients has not been investigated yet. There will also be a need to consider that every APIFat may not share the same characteristics (e.g., adipokine–cytokine secretion) and the effect on different organs may vary [16,17,86,87,95,106].

Further research is needed in this field to elucidate these associations as well as their potential clinical implications.

## Abbreviations

APIFat	abnormal peri-organ or intra-organ fat
BODE index	body mass index (B), degree of airflow obstruction (O), dyspnoea (D) and exercise capacity (E)
COPD	chronic obstructive pulmonary disease
CPAP	continuous positive airway pressure
CRP	C-reactive protein
CV	cardiovascular
EFT	epicardial fat thickness
FEV_1_	forced expiratory volume in 1 s
FVC	forced vital capacity
IL	interleukins
MetS	metabolic syndrome
MI	myocardial infarction
NAFLD	non-alcoholic fatty liver disease
NAFPD	non-alcoholic fatty pancreas disease
OS	overlap syndrome
OSAS	obstructive sleep apnoea syndrome
PAH	pulmonary arterial hypertension
RVSD	right ventricular systolic dysfunction
SAT	subcutaneous adipose tissue
T2DM	type 2 diabetes mellitus
TNF-α	tumour necrosis factor α
VAT	visceral adipose tissue
